# Relationship of vitamin D with the anthropometric indicators and lifestyle of adults. Medellín, Colombia*

**DOI:** 10.15649/cuidarte.2920

**Published:** 2023-12-20

**Authors:** Nubia Amparo Giraldo-Giraldo, Carolina Ramírez-Morales, Yelithza Idárraga-Idárraga, Ángela Restrepo-Moreno, Luz Elena Cano-Restrepo, Susana Pamela Mejía-de-los-Ríos

**Affiliations:** 1 . Universidad de Antioquia. Medellín, Colombia. Food and Human Nutrition Research Group. E-mail: nubia.giraldo@udea.edu.co Universidad de Antioquia Universidad de Antioquia Medellín Colombia nubia.giraldo@udea.edu.co; 2 . Universidad de Antioquia. Medellín, Colombia. E-mail: dcarolina1728@gmail.com Universidad de Antioquia Universidad de Antioquia Medellín Colombia dcarolina1728@gmail.com; 3 . Universidad de Antioquia. Medellín. Colombia. E-mail: yelithza@gmail.com Universidad de Antioquia Universidad de Antioquia Medellín Colombia yelithza@gmail.com; 4 . Corporación para Investigaciones Biológicas. Medellín. Colombia. Medical and Experimental Mycology Group. E-mail: angelares@une.net.co Corporación para Investiga. Biológicas Corporación para Investigaciones Biológicas Medellín Colombia angelares@une.net.co; 5 . Universidad de Antioquia. Medellín. Colombia. Medical and Experimental Mycology Group. E-mail: lula.cano@hotmail.com Universidad de Antioquia Universidad de Antioquia Medellín Colombia lula.cano@hotmail.com; 6 . Corporación para Investigaciones Biológicas (CIB). Medellín. Colombia. Medical and Experimental Mycology Group. E-mail: susanap21@gmail.com Corporación para Investiga. Biológicas Corporación para Investigaciones Biológicas Medellín Colombia susanap21@gmail.com

**Keywords:** Vitamin D, Anthropometry, Life Style, Sunlight, Healthy Volunteers, Vitamina D, Antropometría, Estilo de Vida, Luz Solar, Voluntarios Sanos, Vitamina D, Antropometria, Estilo de Vida, Luz Solar, Voluntários Saudáveis

## Abstract

**Introduction:**

Serum vitamin D levels depend on sunlight, diet, and other factors.

**Objective:**

We aimed to determine serum vitamin D levels and evaluate their relationship with anthropometric indicators and lifestyle habits in apparently healthy volunteers.

**Materials and Methods:**

In this cross-sectional study (n=75), socio-demographic, anthropometric, and lifestyle habit-related data were collected. Serum vitamin D levels were determined with high performance liquid chromatography, food intake was measured by semiquantitative frequency and nutritional status was assessed by anthropometry. Chi-square test and also principal component analysis were used to analyze the relationship between some variables and vitamin D status. Spearman's test was used to determine correlations between quantitative variables.

**Results:**

73% were women and 61% belonged to medium socio-economic level. Median vitamin D intake was 137 (83.1-227.3) IU/day. Based on body mass index (BMI), 44% individuals had overweight/obesity. The 68% exhibited deficient/insufficient vitamin D levels (Hypovitaminosis D). BMI classification and waist circumference (CW) were not related with vitamin D status; however, activities with higher sun exposure were highly related (p = 0.013). Sun exposure time explained variation in component 2 (16.60%), where most of the individuals with normal level were grouped. Sun exposure time was positively correlated with vitamin D status (r = 0.263; p = 0.023).

**Discussion:**

Excess weight and abdominal obesity are not always associated with hypovitaminosis D.

**Conclusions:**

The majority of individuals showed hypovitaminosis D but their status was not related with anthropometric indicators. A Sun exposure time was the only factor positively correlated with vitamin D status.

## Resumen

**Introducción:**

Los niveles séricos de vitamina D dependen de la luz solar, la dieta y otros factores.

**Objetivo:**

Nuestro objetivo fue determinar los niveles séricos de vitamina D y evaluar su relación con indicadores antropométricos y estilos de vida en voluntarios aparentemente sanos.

**Materiales y métodos:**

En este estudio trasversal (n=75) se recogieron datos sociodemográficos, antropométricos y aquellos relacionados con hábitos y estilos de vida. La vitamina D se determinó por cromatografía líquida de alta eficiencia; la ingesta de alimentos, mediante frecuencia semicuantitativa, y el estado nutricional por antropometría. Para analizar la relación entre algunas variables y el estado de la vitamina D se usó la prueba de Chi cuadrado y también el análisis de componentes principales. Se empleó la prueba de Spearman para determinar la correlación entre variables cuantitativas.

**Resultados:**

El 73% eran mujeres y el 61% pertenecían a un estrato socioeconómico medio. La mediana de la ingesta de vitamina D fue de 137 (83,1-227,3) Ul/día. Según el índice de masa corporal (IMC), el 44% de los individuos tenían sobrepeso/obesidad. El 68% mostró deficiencia/insuficiencia de vitamina D (hipovitaminosis D). La clasificación del IMC y la circunferencia abdominal no se relacionaron con el estado de la vitamina D; sin embargo, las actividades con una mayor exposición solar estuvieron altamente relacionadas (p=0,013). El tiempo de exposición solar explicó la variación en el componente 2 (16,60%) donde se agruparon la mayoría de los individuos con niveles normales. El tiempo de exposición solar tuvo una correlación positiva con el estado de la vitamina D (r = 0,263; p = 0,023).

**Discusión:**

El exceso de peso y la obesidad abdominal no siempre se relacionan con la hipovitaminosis D.

**Conclusiones:**

La mayoría de los individuos presentaron hipovitaminosis D, pero su estado no se relacionó con los indicadores antropométricos. El tiempo de exposición al sol fue el único factor que se correlacionó positivamente con el estado de la vitamina D.

## Resumo

**Introdujo:**

Os níveis séricos de vitamina D dependem da luz solar, da dieta e de outros fatores.

**Objetivo:**

Nosso objetivo foi determinar os níveis séricos de vitamina D e avaliar sua relaáo com indicadores antropométricos e hábitos de vida em voluntários aparentemente saudáveis.

**Materiais e métodos:**

Neste estudo transversal (n=75), foram coletados dados sociodemográficos, antropométricos e relacionados a hábitos de vida. Os níveis séricos de vitamina D foram determinados por cromatografia líquida de alto desempenho, a ingestáo de alimentos foi medida por frequencia semiquantitativa e o estado nutricional foi avaliado por antropometria. O teste do qui-quadrado e a análise de componentes principais foram utilizados para analisar a relaáo entre algumas variáveis e o estado da vitamina D. O teste de Spearman foi usado para determinar as correlates entre as variáveis quantitativas.

**Resultados:**

73% eram mulheres e 61% pertenciam a um nivel socioeconómico médio. A ingestáo média de vitamina D foi de 137 (83,1-227,3) UI/dia. Com base no índice de massa corporal (IMC), 44% dos indivíduos tinham sobrepeso/obesidade. Os 68% apresentaram níveis deficientes/insuficientes de vitamina D (hipovitaminose D). A classificaáo do IMC e a circunferencia da cintura (CW) náo foram relacionadas ao status da vitamina D; entretanto, as atividades com maior exposiáo ao sol foram altamente relacionadas (p = 0,013). O tempo de exposiáo ao sol explicou a variado no componente 2 (16,60%), onde a maioria dos indivíduos com nível normal foi agrupada. O tempo de exposiáo ao sol foi positivamente correlacionado com o status de vitamina D (r = 0,263; p = 0,023).

**Discussao:**

O excesso de peso e a obesidade abdominal nem sempre estáo associados com a hipovitaminose D.

**Conclusóes:**

A maioria dos indivíduos apresentou hipovitaminose D, mas seu status náo foi relacionado com indicadores antropométricos. O tempo de exposiáo ao sol foi o único fator positivamente correlacionado com o status da vitamina D.

## Introduction

Vitamin D is a micronutrient of great importance to human health, it has a rol in bone homeostasis but also it functions as a hormone affecting physiological processes such as secretion and effectiveness of insulin, regulation of the renin-angiotensin-aldosterone system, immune system activation and cell cycle control and apoptosis. Moreover, vitamin D deficiency is related to a higher risk of diabetes mellitus, cardiovascular, oncologic, infectious, and autoimmune diseases^1^.

The main source of vitamin D is exposure to ultraviolet B (UV-B) rays from sunlight whereas foods such as salmon, tuna, eggs, and fortified foods like milk, margarine, and processed cereals for breakfast represent minor sources^2^.

Factors related to synthesis, lifestyle habits, obesity, malabsorption, hepatic and renal diseases and some drugs could, promote a deficit of this vitamin. Vitamin D deficiency can lead to osteomalacia and osteoporosis, there is also an increased risk of bone pain, bone fractures, muscle pain and muscle weakness. In older adults, severe deficiency may contribute to an increased risk of falls^3^.

The medical literature states that overweight or obese individuals have lower vitamin D levels. The mechanisms underlying this relationship are correlated to vitamin D retention in the adipose tissue, an increase in catabolism due to the localized action of the 24-hydroxylase, or a decrease in hepatic hydroxylation. In addition, there could be other underlying reasons pertaining to the lifestyle habits of overweight individuals, such as fewer outdoor activities and using clothes that cover the body more^4^.

It is estimated that almost a billion people globally suffer from a deficiency of vitamin D. In the United States, Canada, Mexico, Europe, and Australia, deficiency/insufficiency are between 30% and 50% of the population, including adults and children^2^. This situation is even more severe in older adults in the United States and Europe, where 100% people are reported to have vitamin D deficiency^5^. In a systematic review, the prevalence of vitamin D deficiency was assessed in a healthy population from all ages in South America and the Caribbean, reporting deficient levels of this vitamin (<20 ng/ mL) in 20%-40% population^6^. In Colombia, a vitamin D deficiency has been observed in patients with osteoporosis and two-thirds of postmenopausal women could have low levels of vitamin D^7^. However, in Medellín Colombia, there are no studies in healthy adults that evaluate the serum levels of vitamin D and relate them to anthropometric indicators and lifestyles.

The aim of this study was to determine the serum levels of vitamin D and explore its relationship with anthropometric indicators and lifestyle habits.

This study is relevant from public healthcare perspective because vitamin D deficiency/insufficiency implies long-term high morbidity and mortality in chronic diseases, which constitute health problems in Colombia. Therefore, it is necessary to focus the efforts in solving this issue and to plan preventive and intervention strategies.

## Material and Methods

### Type of study and population

An analytical cross-sectional study was conducted on 75 adult volunteers, aged 18-60 years from Universidad de Antioquia and the Corporación para Investigaciones Biológicas (CIB); in the city ofMedellin, Colombia. The study was done at these two institutions because the researchers work there and it facilitated the recruitment of participants. The subjects had to be apparently healthy at the time of their recruitment into the study, without any suggestion of liver, kidney autoimmune or gastrointestinal disease.

Individuals who used multivitamin supplements, medication, psychotropic drugs and who had an alcohol intake of >180 mL/day were excluded. Furthermore, pregnant women, lactating women, and smokers were also excluded.

For recruitment of participants, we set up an open call at the above mentioned institutions. E-mails were sent with the project's briefing, explaining relevant dates.

All volunteers were assessed regarding the inclusion criteria. A peripheral blood sample (12 mL) was drawn from 125 participants to determine vitamin D serum levels.

In the second call, 75 participants attended. They were subjected to a personalized interview with nutrition students from the last year inquiring sociodemographic, anthropometric data inquiring socio demographic, anthropometric, lifestyle habits (sun exposure/minutes per day, use of sunscreen, color of regularly used clothes, and sun exposure of limbs), skin type, and vitamin D intake. The sample was collected for six months. For the analysis, the participants were grouped according to their age as follows 18-25, 26-35 and >35 years; subjects were classified into these three age categories because they were grouped by tertiles to make the distribution more even. Three categories were established according to their socio-economic level: high (levels 5 and 6), medium (levels 3 and 4), and low (levels 1 and 2). Furthermore, they were categorized into high and low exposure according to their employment type depending on whether they conduct activities in open or closed spaces.

Additionally, lifestyle habit variables were also categorized. The use of sunscreen was determined according to the level of sun protection factor (SPF): appropriate protection (SPF >40), low protection (SPF10 and SPF30), and no protection. The exposure to sunlight of the limbs was grouped as high (>3 limbs), low (1 or 2 limbs), and none.

Skin color was determined following Von Luschan's and Fitzpatrick's chromatic scales. This color palette scale was compared with the internal area of the forearm and characterized with a number, where number 1 corresponded to light skin and number 36 to dark skin^8^. The latter scale was used during a personalized interview to obtain information about sunburn and the ability to tan, estimating the skin's tolerance to ultraviolet (UV) light^9^. Finally, we considered the results from both scales and categorized the participants as follows: light sensitivity (phototypes I and II), normal sensitivity (phototypes III and IV), and tolerance to sunlight (phototypes V and VI).

### Dietary Data

For the intake questionnaire, food sources of vitamin D published by Zuluaga N et al. natural sources such as various types of fish, egg yolk and mushrooms were included, as well as fortified foods such as milk, yogurt, margarine and breakfast cereals^1^. Additionally, we evaluated several nutritional content labels on different products from supermarkets in the city of Medellin with the aim of including foods fortified with vitamin D. The questionnaire included 20 foods and it was tested on students from the Universidad de Antioquia. To quantify the participant's intake of each food, we used a quantitative frequency method for the list of 20 foods. Food items were separated into modules on the basis of portion size and weight (in grams) to quantify intake of solid foods. For liquid foods, we used photographs of glasses, mugs, bowls, and spoons to estimate the quantity in milliliters. The data obtained were recorded in the food-frequency instrument for consumption of food sources of vitamin D. Subsequently, the amount of vitamin D corresponding to the 20 foods was entered in an Excel sheet and the frequency of food intake (daily, weekly, or monthly) of each participant was recorded. Next, the daily average intake of vitamin D was calculated.

### Anthropometric measurements

*We* conducted an anthropometric assessment followed Lohman's method^10^. The body weight was measured in kg using a TANITA digital scale (sensitivity of 0.1 kg) (model HD-313, TANITA, Arlington Heights, IL, US); height was measured in cm with a SECA wall stadiometer (sensitivity of 0.1 cm) (model 206, SECA, Hammer Steindamm, HSV, BRD); CW was measured between the last rib and the iliac crest using a SECA metric measuring tape (sensitivity of 0.1 cm) (model 201, SECA, Hammer Steindamm, HSV, BRD).

We classified the participants based on their BMI according to the categories stated by the World Health Organization as follows: appropriate weight, 18.5-24.9 kg/m2; overweight, 25-29.9 kg/m2; and obese >30 kg/m2^11^. To establish the abdominal obesity related to CW, we used the following indications recommended by the International Diabetes Federation: for men no risk (<90 cm), high risk (90-102 cm), and very high risk (>102 cm); and for women no risk (<80 cm), high risk (80-88 cm), and very high risk (>88 cm)^12^.

### Vitamin D measurement

To determine vitamin D serum levels, we quantified 25-hydroxivitamin D3 (25-(OH)D3) using high performance liquid chromatography (HPLC) with ultraviolet (UV) detection model 1200, Agilent Technology, Santa Clara, CA, US^13^. The 25-(OH)D3 is considered the most abundant metabolite precursor of vitamin D and best reflects the level status of this vitamin in the body^14^. For the classification of vitamin D levels, we followed the Endocrine Society Clinical Practice Guideline: normal (30-150 ng/mL), insufficient (21-29 ng/mL), deficient (<20 ng/mL)^15^. We used only two classifications for their analysis, normal > 30 ng/mL and low values < 30 ng/mL, in which both the deficient and the insufficient are found.

### Statistical analysis

The statistical analyses was performed with the Statistical Package for Social Sciences software, SPSS (version 24.0, SPSS Inc, Chicago, IL, USA). The database was stored in Mendeley data^16^. The quantitative variables were described by central and dispersion tendency measures. Qualitative variables were described by frequencies and percentages. We used Chi-square or Fisher's test to analyze the association among status of vitamin D and socio-demographic and lifestyle habits variables. The Mann Whitney's U test was used to determine the relation among average BMI and waist circumference with the vitamin D classification. In either case an exploratory analysis of the variables was conducted by a principal components analysis (PCA) with the help of Rstudio; in addition, Packages FactoMineR and Factoextra were used for the analysis. To establish a correlation between the vitamin D serum levels and the quantitative variables such as age, BMI, vitamin D intake, and sun exposure time, we used the ggplot2 package in Rstudio. The defined significance level was p <0.05. To confirm the correlations between variables, we performed linear regressions of the significant variables using the Stats package in Rstudio. The charts were created using GraphPad Prism (version 5, GraphPad Ind, San Diego, CA, US) and Rstudio (RStudio Inc, Boston, MA, US).

### Ethical considerations

According to the principles of the Declaration of Helsinki and the Ministerio de Salud de Colombia in the Section 11, Resolution Number 008430 of October 1993, this research is classified as minimal ethical risk. The CIB's bioethics committee ratified and approved the methods for this study in the minutes # 101. Prior to beginning the study, all participants signed a consent form.

## Results

### Participants' characteristics

The median aged was 30 years, interquartile range (IQR 24 - 41). Seventy-three percent of the participants were women n (55), 61.33% n (46) belonged to the medium socio-economic level, 53.27% n (46) were aged 18-35 years, and 70.33% n (63) had normal sun sensitivity (Table 1). According to the BMI, 44.00% of the individuals were overweight or obese, 29 (38.60%) were overweight and 6 (8.00%) obese. According to the waist circumference, 25.33% n (19) of the individuals had abdominal obesity (Table 2).

Median vitamin D intake was of 137 IU (IQR 83.1-227.3) and median sun exposure time was 30 min (IQR 15-60).

Among subjects >35 years of age, 80.80% n (21) had deficiency / insufficiency levels vitamin D. Moreover, 69.10% n (38) of women and 65.00% n (13) of men showed deficient/insufficient levels. Interestingly, individuals who belonged to the high socio-economic level showed a higher proportion of serum vitamin D deficiency/insufficiency (78.6%), n (11) whereas the ones who belonged to lower socio-demographic levels showed lesser deficiency/insufficiency (53.30%) n (8). Regarding individual's employment type and their exposure to sunlight, it was found that those with a higher sun exposure (60.00%) n (9) exhibit normal serum vitamin D levels, whereas of those with lower exposure to sunlight, 25.00% n (15) had normal levels (p=0.013). In the group using darker clothes, 80.00% n (24) of individuals showed deficient/insufficient vitamin D. The majority of the individuals with normal levels of vitamin D (62.50%) n (5), had more number of limbs exposed to the sunlight (Table 1).

**Table 1:** Differentiation between groups according to classification of vitamin D levels and socio-demographic and lifestyle variables

Variable	Groups				
	Deficient/insufficient	(n=51)	Normal (n = 24)		Chi2
	n	Frequency %	n	Frequency %	^*p*^
Age group					0.16
18-25	17	68.00	8	32.00	
26-35	13	54.20	11	45.80	
>35	21	80.80	5	19.20	
Sex					0.47
Women	38	69.10	17	30.90	
Men	13	65.00	7	35.00	
Socio-economic level					0.33*
High	11	78.60	3	21.40	
Medium	32	69.60	14	30.40	
Low	8	53.30	7	46.70	
Employment					0.01
High sun exposure	6	40.00	9	60.00	
Low sun exposure	45	75.00	15	25.00	
SPF level (Sun Protection Factor)					0.55*
Adequate	26	65.00	14	35.00	
Low	4	57.10	3	42.90	
No protection	21	75.00	7	25.00	
Clothes color					0.14
Dark	24	80.00	6	20.00	
White	5	50.00	5	50.00	
Mixed	22	62.90	13	37.10	
Limb exposure					0.87*
High	3	37.50	5	62.50	
Low	47	72.30	18	27.70	
None	1	100.00	0	0.00	
Skin phototype					0.42*
Sunlight sensitivity	7	87.50	1	12.50	
Normal sensitivity	41	65.10	22	34.90	
Tolerance to sunlight	3	75.00	1	25.00	

Results are presented by row *Fisher's exact test

Statistical significance of difference is calculated between subjects with and without vitamin D deficiency.

### Relationship between anthropometric measurements and vitamin D serum levels

According to anthropometric measurements, 60.60% n (20) of overweight/obesity participants and 57.90% n (11) of high CW (abdominal obesity), presented deficiency/insufficiency vitamin D. Despite the fact that hypovitaminosis D was more frequent in individuals with excess weight and abdominal obesity, there were no statistically significant differences between the nutritional state and vitamin D serum level classification when Chi-square test was used (Table 2).

**Table 2:** Differentiation between groups according classification of vitamin D levels and anthropometric parameters

Variable	Groups				
	Deficient/insufficient (n=51)		Normal (n = 24)		Chi2
	n	Frequency %	n	Frequency %	^*p*^
Waist circumference					0.20
High (abdominal obesity)	11	57.90	8	42.10	
Normal	40	71.40	16	28.60	
BMI					0.34*
Adequate	29	72.50	11	27.50	
Thinness	2	100.00	0	0.00	
Overweight/obesity	20	60.60	13	39.40	

Results are presented by row *Fisher's exact test

Statistical significance of difference is calculated between subjects with and without vitamin D deficiency.

When average BMI and CW were analyzed in relation with vitamin D status using Mann-Whitney U-test, no statistically significant differences were found in either case.

### Multivariate analysis

**Figure 1:**
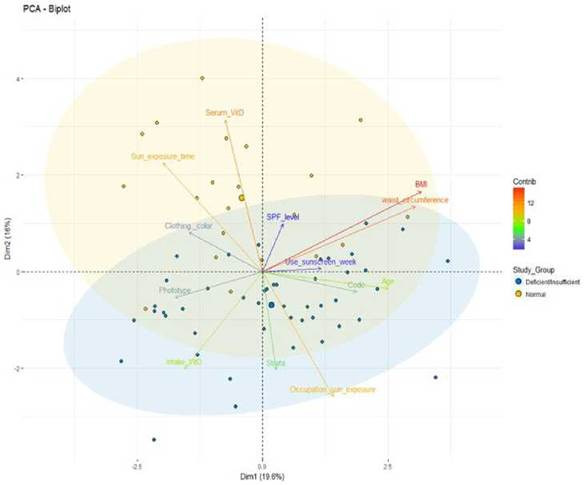
A principal components analysis

The PCA was conducted with the data of 69 participants. Six participants identified as outliers were excluded. PCA showed that components 1 and 2 explained the 36.20% variance. Sunlight exposure and vitamin D serum levels are the parameters which can explain the greater variance of component 2 (16.60%), where most of the individuals classified as normal are present. This allows for a distinction of this group from the deficient/insufficient one (Figure 1). Employment with outdoor activities and vitamin D intake are the parameters that can better explain the variance of component 1 (19.60%) in which most of the individuals with deficient/insufficient serum levels of vitamin D can be found, allowing a separation from the group with normal vitamin D levels. None of the other factors analyzed were significant in this data set (Figure 1).

The dots show the separation between the two groups studied according to the socio-economic variables and other factors related to vitamin D: deficient/insufficient (blue) and normal (yellow). The color scale of each arrow and the name of each variable represent how much does each variable contribute to the main component, red being the one that contributes the most and blue being the one contributing less (see color scale to the right).

**Figure 2:**
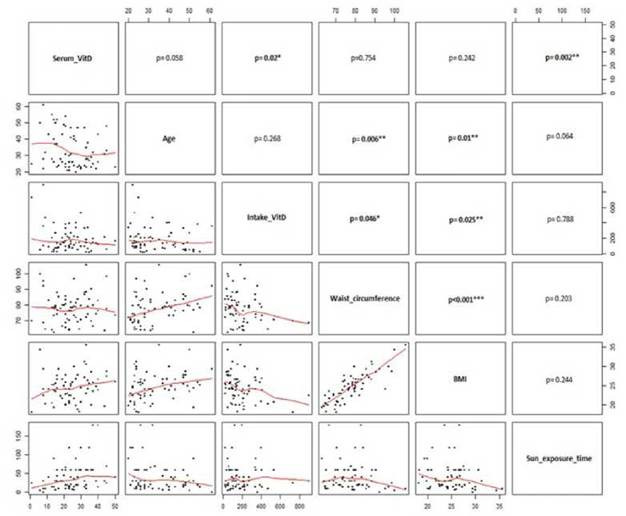
Correlation between anthropometric variables and factors related to vitamin D levels.

To establish the correlation between vitamin D serum levels with different variables such as age, vitamin D intake, waist circumference, BMI, and time of sunlight exposure, we used Spearman's test. A significant positive correlation between vitamin D serum levels and time of sunlight exposure (p= 0.002); age and CW (p= 0.006); age and BMI (p= 0.01); and CW and BMI (p= 0.001) were observed (Figure 2).

The lower panels show the distribution of raw data (black dots) and the red line indicates the smoothened trend. The upper panels show the p values of the different correlations. The correlations indicating a relevant p value are indicated with boldface (*p < 0.05; **p < 0.01; y *** p < 0.001).

**Figure 3:**
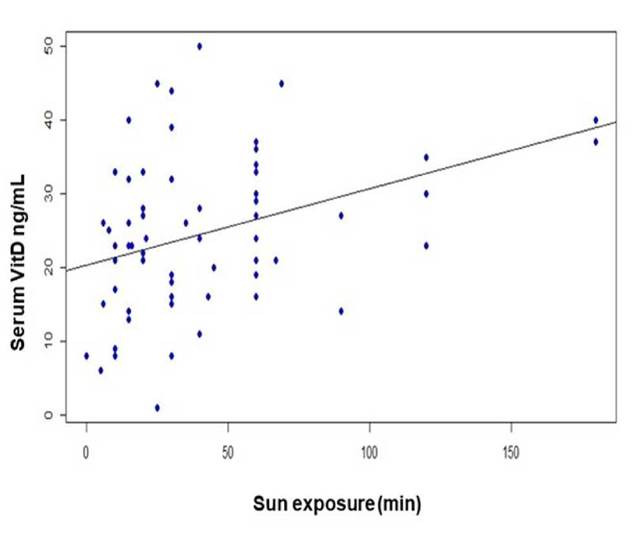
Linear regression between Vitamin D serum levels and sun exposure time (min)

To verify the correlation results, we individually performed linear regression of the variables of time of sunlight exposure (in minutes) with vitamin D serum levels, showing a positive correlation with sun exposure (R2= 0.136 p= 0.002) (Figure 3).

## Discussion

Although this study was conducted on apparently healthy adults, the proportion of individuals with deficient/insufficient serum vitamin D levels was surprisingly high (68%), despite the city of Medellin, Colombia, being at a tropical latitude (6°13'1" N, 75°34'1'' W). The proportion of individuals with deficient/insufficient vitamin D was higher than those reported in Saudi Arabia (51.7%)^17^ and Singapore (42%)^18^; our results were similar to those reported in Brazil (64.2%)^19^, but lower than those reported in Gansu province, China (93,7%)^20^.

These differences could be attributed to techniques used to determine vitamin D serum levels; some studies used chemiluminescence immunoassay (CLIA) test^14^, whereas others used tandem mass spectrometry coupled with liquid chromatography (MS/MS-LC)^13^, and in this study we used HPLC. MS/ MS-LC and HPLC offer a higher sensitivity in 25(OH)D3 metabolite detection. In addition, the system of classifying individuals on the basis of vitamin D serum levels was different in one of these studies^17^.

We did not find any significant differences between BMI or CW between individual with normal levels and those with deficient/insufficient levels of vitamin D according with other studies in adults^21^^,^^22^. Khader et al. in Jordan used sample of adults aged >18 years and reported no significant statistical association between vitamin D levels and the metabolic syndrome components such as CW and BMI^23^. Khan et al. also failed to find any significant association between vitamin D status and obesity ^24^. Conversely, Rocha et al., in Brazil, found an association between high BMI and deficient vitamin D levels^19^, and Cartier et al. found that 25(OH)D3 negatively correlated with BMI^25^.

Recently Mansouri et al, in a study with professors, concluded that serum 25(OH)D concentrations were inversely associated with general and abdominal obesity either before or after controlling for potential confounders^26^. Similar findings were reported by Hajhashemy et al in a systematic review and meta-analysis of epidemiologic studies, finding that serum vitamin D level was inversely associated with risk of abdominal obesity in adults, in a dose-response manner^27^. These differences may be due to the fact that both studies used cut-off points to classify obesity, different from ours. In addition, in the meta-analysis they did not include in the Forest plots, studies from South American countries, which have different lifestyle habits.

The average vitamin D intake in this study was 182.4 ± 138.2 IU/day. This value is higher than the one reported by Martini et al. (130.8 ± 66.0 IU/day in adult men and 108.8 ± 55.6 IU/day in adult women)^28^. These differences could be attributed to the diversities in the methodology used for intake quantification. The Brazilian study^19^ used a 24-hour reminder, whereas this study recorded the intake frequency with a list of 20 foods sources of vitamin D.

The Institute of Medicine (IOM) in the United States revised the requirement of vitamin D and established that the Estimated Average Requirement (assuming minimum sun exposure) for people aged 1-70 years is 400 IU/day^29^. According to the results obtained from the studies mentioned before and despite the differences in the intake quantification methods, vitamin D consumption is lower than that recommended by the IOM and by the Energy and Nutrients Intake Recommendations for Colombian population^30^. Thus, there should be food fortification policies or campaigns on minimum sunlight exposure requirements so that people will achieve adequate levels of vitamin D. Regarding employment types wherein activities are conducted outdoors, this study confirmed that there is a higher proportion of individuals with normal levels of vitamin D (2:1) whose employment required a higher sunlight exposure; on the contrary, individuals with low sunlight exposure presented a higher proportion (3:1) of deficiency/insufficiency of vitamin D. This finding agrees with that reported in a systematic review stating that employees working in shifts, health professionals, and others working in close spaces have a greater risk of having a vitamin D deficiency owing to their lifestyle that leads to less exposure to UVB rays^31^.

Serum levels of 25(OH)D3 are a good indicator of accumulated sun exposure and dietary intake of vitamin D; estimation using this metabolite is widely known as the gold standard technique of estimating vitamin D status. UVB rays are the main source of vitamin D for the majority of individuals; however, adverse effects of UVB rays are frequently documented, particularly for people with fair skin. Thus, there has been an increase in the use of photo protectors, primarily sunscreens. This negatively affects vitamin D status. Furthermore, individuals with highly pigmented skin have a poor vitamin D status due to photoprotection provided by melanin^2^. In our study, sun exposure time was the only factor that was positively correlated with vitamin D serum levels, implying that UVB rays are the best source of the vitamin. In Spain, hypovitaminosis D was highly prevalent among primary health care users, and significantly higher in winter as compared to summer^32^.

## Conclusions

The proportion of healthy individuals with deficient/insufficient vitamin D serum levels was quite high, and it was not associated with anthropometric indicators. Sunlight exposure time was the only factor positively correlated with these values. Although vitamin D deficiency/insufficiency does not necessarily indicate an overt disease, it implies a risk for chronic diseases; thus, it warrants immediate attention.

Vitamin D intake was lower than the recommended value, and this should alert the population of the risk it implies for their health.

A merit of our study is determining 25(OH)D3 levels using HPLC coupled with UV detection, which is a physical non-immunologic method of detection with a higher precision than that of the immunoassay.

Limitations: It is a cross-sectional study that does not measure exposure to a factor and the cause-effect relationship is not verifiable. There may be selection biases because the recruitment was done with volunteers and the population sample was not representative of the city. The measurement of vitamin D intake was made with a frequency of food consumption and there may be other more precise methods such as 24-hour recall on several days a week.
